# Characteristics of the Fractional Amplitude of Low-Frequency Fluctuation in Ocular Hypertension Patients: A Resting-State fMRI Study

**DOI:** 10.3389/fmed.2021.687420

**Published:** 2022-04-08

**Authors:** Ying Liang, Yi-Cong Pan, Hui-Ye Shu, Xue-Mei Chou, Qian-Min Ge, Li-Juan Zhang, Qiu-Yu Li, Rong-Bing Liang, Han-Lin Li, Yi Shao

**Affiliations:** Department of Ophthalmology, The First Affiliated Hospital of Nanchang University, Jiangxi Province Ophthalmology Institute and Ocular Disease Clinical Research Centre, Nanchang, China

**Keywords:** ocular hypertension, resting state functional magnetic resonance imaging, fractional amplitude of low-frequency fluctuation, left anterior cingulate cortex, left precuneus

## Abstract

**Background:**

The fractional amplitude of low-frequency fluctuation (fALFF) method has been underutilized in research on the pathogenesis and clinical manifestations of ocular hypertension (OH).

**Purpose:**

This study uses resting state functional magnetic resonance imaging (rs-fMRI) and fALFF to investigate the nature of spontaneous brain activity in OH patients and the relationship, if any, between changes in activity and clinical features.

**Materials and Methods:**

A total of 18 subjects (9 females and 9 males) with ocular hypertension (OH) and 18 healthy controls (HCs) matched for gender, age, and educational level were recruited to this study. All participants underwent an rs-fMRI scan, and spontaneous brain activity was assessed using the fALFF method. Receiver operating characteristic curves were plotted to investigate differences between OH and HC groups.

**Results:**

The fALFF values of OH patients were significantly higher in the left precuneus lobe (LP), compared with the same region in controls (*P* < 0.05). Conversely, values in the left anterior cingulate lobe (LAC), were significantly lower (*P* < 0.05) in OH than in controls. However, no significant association was found between the mean fALFF values and clinical characteristics in either brain area.

**Conclusion:**

High spontaneous activity in two brain areas may reflect neuropathological mechanisms underpinning visual impairment in OH patients.

## Introduction

Ocular hypertension (OH) is a condition in which intraocular pressure (IOP) is >21 mmHg (1 mmHg = 0.133 kPa) but without glaucomatous fundus appearance or optic nerve damage (1). Studies have found that for every 1 mmHg increase in IOP, the risk of glaucoma will increase by 12%, and that for every 1 mmHg interocular difference in IOP the risk of OH transforming into glaucoma increases by 21% (2). Although not all people with OH will develop glaucoma (3), Elevated IOP has always been considered the main risk factor for the progression of glaucoma (4), and it is also the only known modifiable risk factor in the disease (5), about 9.5% will develop glaucomatous optic nerve damage in their lifetime (6).

Generally, OH is considered to be a pre-glaucoma state (7, 8). Worldwide, glaucoma is the most common cause of irreversible blindness, and it is anticipated that by 2040, 111.8 million of the global population will have this disease (9). Since IOP is positively correlated with the development of glaucoma an understanding of the pathogenesis of OH is important to guide strategies for the prevention and treatment of glaucoma, a significant public health problem.

Many studies have investigated the nervous system in eye disease. For example, magnetic resonance imaging (MRI) has been used in research on the pathogenesis of glaucoma (10–20), and found that glaucomatous damage extends not only to the visual cortex but also to other parts of the central nervous system (21–23). This provides an important basis for further research on the neurological characteristics of patients with ocular hypertension. Resting state functional magnetic resonance imaging (rs-fMRI) is a relatively new imaging technique allowing efficient assessment of brain function in healthy people and patient populations (24). It is simple and easy to operate, non-invasive, with high spatial and temporal resolution. The resting state signals and functional responses can be used to assess brain activity and tissue morphology in non-task states and to analyze disease mechanisms affecting brain tissue (25, 26). Research methods based on rs-fMRI such as functional connectivity (Fc), regional homogeneity (ReHo), voxel-based morphometry (VBM),amplitude of low-frequency fluctuation(ALFF), and fractional ALFF (fALFF) can explore abnormal functional changes in brain regions related to the occurrence and development of diseases. A study on OH found that abnormal regional activity appeared in patients with high-tension glaucoma (27). ALFF method refers to the average value of the square root of the power spectrum of the BOLD signal in the low frequency range (0.01-0.08 Hz). It is defined as a practical method of rs-fMRI to measure the strength of intensity of low-frequency oscillations (LFOs) and the spontaneous brain activity of the subject in a resting state (28). It has been widely applied research of ophthalmologist diseases such as high myopia (HM) (29), glaucoma (30, 31), optic neuritis (OP) (32), diabetic retinopathy (DR) (33), retinal vein occlusion (RVO) (34), thyroid-associated ophthalmopathy (TAO) (35), retinitis pigmentosa (RP) (36), comitant exotropia (CE) (37). However, because the accuracy of ALFF is negatively affected by non-neural activities such as movement, respiratory and cardiac noise. The fALFF was proposed by Zou et al. (38), it is the sum of the power spectrum amplitudes in the entire frequency range (0–0.25 Hz) which is divided by the amplitude in the frequency range of 0.01–0.08 Hz. It suppresses the non-specific signal components in the rs-fMRI response spectrum, so it has higher sensitivity and specificity in reflecting the changes of BOLD signal (39). The fALFF method can not only widely analyze various eye diseases (36, 37, 40–45), but also used to assess different diseases of other organs and systems (46–52) as an advanced tool (see Table 1). In this way, the fALFF method has great potential for advancing the analysis, diagnosis and treatment of other ophthalmic diseases. Therefore, through the qualitative and quantitative information it provides, rs-fMRI was combined with fALFF in the present study to observe spontaneous brain activity in the resting state and explore the neurophysiological mechanism of OH.

**Table 1:** fALFF method applied in ophthalmologic and other diseases.

	**References**	**Disease**
**fALFF method applied in ophthalmologic and other diseases**		
Ophthalmologic	Li et al. (41)	Primary Open-Angle Glaucoma (POAG)
diseases	Huang et al. (36)	Retinitis Pigmentosa (RP)
	Fang et al. (44)	Monocular Blindness (MB) (44)
	Chen et al. (37)	Comitant Exotropia (CE)
	Li et al. (40)	Normal-tension Glaucoma (NTG)
	Wang et al. (42)	Primary Angle-Closure Glaucoma (PACG)
	Zhang et al. (43)	Neovascular Glaucoma (NVG)
	Feng et al. (45)	Congenital Blindness (CB)
Other diseases	Chen et al. (46)	Acute Incomplete Cervical Cord Injury (AICC)
	Seidel et al. (49)	Anorexia Nervosa (AN)
	Ning et al. (50)	Bipolar disorder (BD)
	Yang et al. (47)	Alzheimer's disease (AD)
	Puche et al. (48)	Parkinson's disease (PD)
	Ao et al. (51)	Irritable Bowel Syndrome with Diarrhea (IBS-D)
	Piao et al. (52)	Systematic Lupus Erythematosus (SLE)

## Materials and Methods

### Subjects

From November 2019 to September 2020, 18 OH patients (9 males and 9 females, mean age 51.18 ± 5.22 years; age range 20–60 years) and 18 HCs (9 males and 9 females; mean age 50.36 ± 5.42 years; age range 20–60 years) were recruited from the Ophthalmology Department of the First Affiliated Hospital of Nanchang University. The inclusion criteria of the OH group [in accordance with the Guideline Development Group (40)] were: (1) age 20–60 years; (2) right-handed; (3) junior high school education or above; (4) without mental illness such as depression, or drug abuse and other mismatched recipients; (5) no history of eye surgery such as laser treatment; (6) no other eye disease such as amblyopia or retinal macular disease; (7) open anterior chamber angles; (8) IOP > 21 mmHg in at least one eye without treatment; (9) no typical glaucomatous visual field defect (examined by the Humphrey 24-2 SITA standard program); (10) no nerve fiber layer defect detected by optical coherence tomography (Carl ZEISS Meditec, Inc. Cirrus HD-OCT, model 4000); (11) no typical glaucomatous changes at the optic nerve head such as enlargement of the glaucoma optic cup or narrowing of the disc edge; (12) no secondary cause of IOP increase such as ocular trauma, uveitis, or cataract; (13) no contraindication to MRI (e.g., no pacemaker or other metal foreign body).

The inclusion criteria of the healthy control group (HC) were identical, with the addition of the following: (1) best corrected visual acuity (BCVA) ≥ 1.0 and IOP in the range 10–21 mmHg; (2) no deformity on MRI examination of brain parenchyma;

The study was approved by the Medical Ethics Committee of the First Affiliated Hospital of Nanchang University and followed the tenets of the Helsinki declaration. Signed informed consent was obtained from all subjects.

### rsfMRI Recording Procedure

Each MRI scan was of approximately 20 min' duration and was conducted by two experienced imaging doctors using a Siemens 3.0T Trio A Tim MRI scanner (Trio; Siemens healthineers). The instrument parameters were based on: In the axial direction, use the three-dimensional damage gradient recall sequence and set (repetition time = 1,900 ms, echo time = 2.26 ms, thickness/gap = 1.0 m/0.5 mm, acquisition matrix = 256 × 256, field of view = 250 × 250 mm, flip angle = 9°) excluded brain space-occupying diseases and obtained 176 images. Then, we use (repetition time = 2,000 ms, echo time = 30 ms, thickness/gap = 4.0 /1.2 mm, acquisition matrix = 64 × 64, field of view = 220 × 220 mm, flip angle = 90°) obtained 240 brain functional magnetic resonance images. Finally, 29 axial slices with gradient echo planar imaging pulse sequences covering the entire brain were obtained.

### Analysis of fMRI Data

On the Statistical Parametric Mapping (SPM; http://www.fil.ion.ucl.ac.uk/spm; The MathWorks, Inc.) platform, performing preliminary processing on the obtained rsfMRI data according to the following steps: (1) Eliminating the first 10 volumes to improve the accuracy of the data, then using the Data Processing Assistant for rs-fMRI (DPABI, http://www.restfmri.net) to perform inter-layer time correction and head movement correction processing on the remaining 230 volumes; (2) Normalizing all fMRI images to meet the standard Montreal Neurological Institute (MNI) space, using 3 × 3 × 3 mm^3^ to resample; (3) Using a Gaussian kernel of 4-mm full-width at half-maximum to smooth all functional images; (4) Performing band-pass filtering (band-limit range 0.01 – 0.08 Hz) on the time series of each voxel to reduce low-frequency drift and high-frequency noise (e.g., cardiac and respiratory noise).

### fALFF Methodology

Briefly, we have realized the process of converting the filtered time series of the obtained voxels into the frequency domain through the fast Fourier transform program. First, the resting state functional magnetic resonance data analysis (REST) toolkit (43) was used to calculate the ALFF value (The average square root of the power spectrum) of the voxels. Then, calculated the fALFF value (the ratio of the power in the frequency band to the power in the entire detection frequency range) of all voxels in the frequency bands of 0.01–0.1, 0.01–0.027, and 0.027–0.073 Hz of the whole brain. Finally, in order to reduce the data bias caused by inter-subject variability, we corrected the fALFF value based on the average fALFF value of whole brain.

### Statistical Analysis

The chi square test was used to compare clinical categorical variables between the HC and OH groups and an independent sample *t*-test was used for continuous data. SPSS software (version 26.0; IBM Corp.) was used for both types of analysis. Data were expressed as mean and standard error of the mean (SEM), and *P*-values <0.05 were considered statistically significant. Voxel level differences between the HC and OH groups were calculated using the REST toolkit with an independent sample *t*-test (53), and its statistical threshold was set to P <0.05. Gaussian Random Field (GFR) theory were used for calibrations (two-tailed, voxel-level *P* < 0.01, cluster-level *P* < 0.05).

### ROC Analysis

The fALFF values of different brain regions were compared using ROC curves, and the correlations between mean fALFF values and related behaviors were analyzed using Pearson's correlation. Data were plotted and correlations calculated using GraphPad Prism (version 8.0; GraphPad Software, Inc).

## Results

### General Analysis

**Figure 1:**
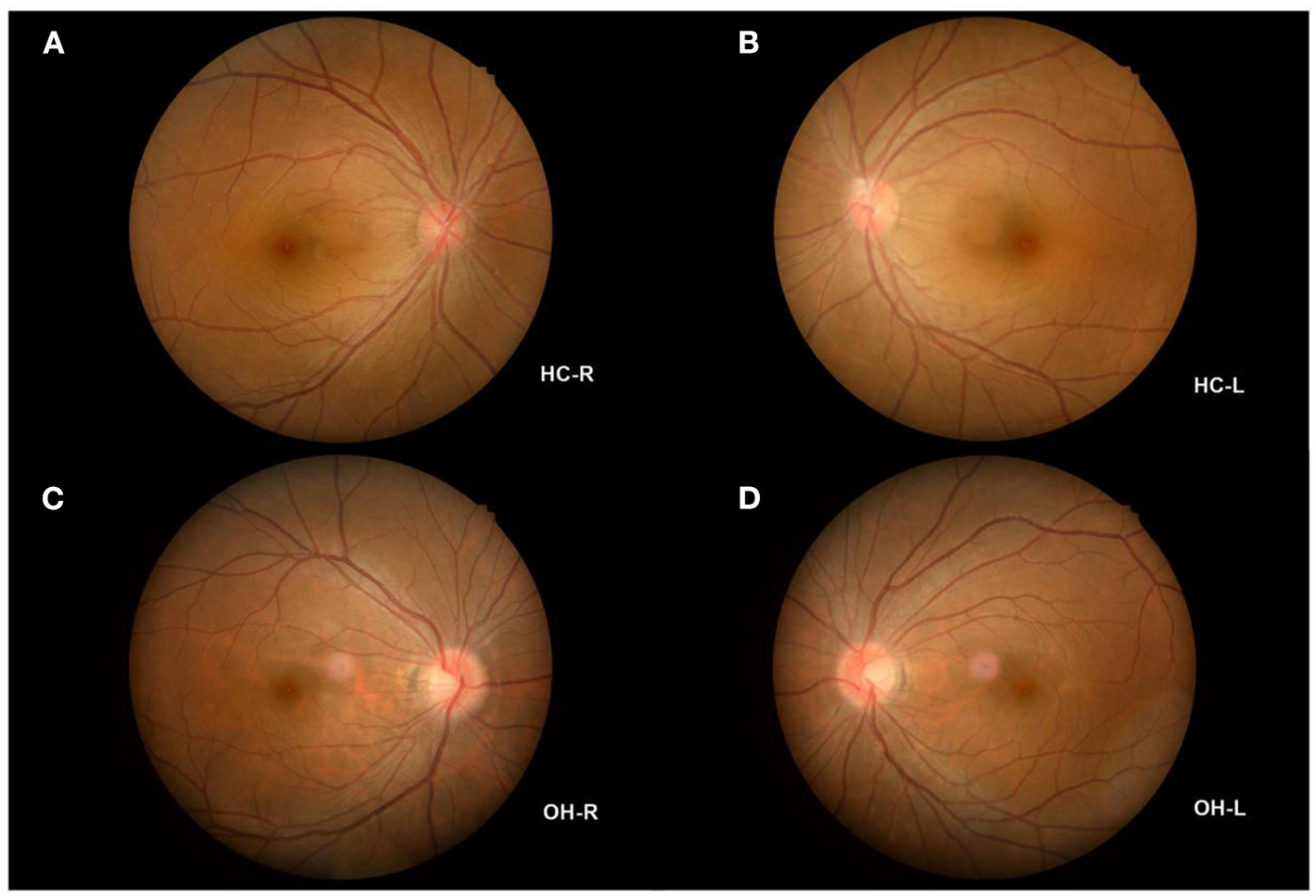
Example of OH and HC observed using a FC. **(A)** The fundus of HC-R; **(B)** The fundus of HC-L; **(C)** The fundus of OH-R; **(D)** The fundus of OH-L. OH, ocular hypertension; HC, healthy control; R, right; L, left; FC, fundus camera.

No statistically significant differences were found between the two groups in terms of gender, age, or inertia (*P* > 0.05). Monocular BCVA, IOP, optic disc color, and cup-to-disk ratio were also statistically similar between the two groups (*P* > 0.05). Mean duration since diagnosis in the OH group was 1.12 ± 0.32 years (Table 2; Figure 1).

**Table 2:** Demographics and behavioral results of OH and HCs groups.

	**OH**	**HC**	***T*-value**	***P*-value**
Male/female	9/9	9/9	N/A	>0.99
Age (years)	51.18 ± 5.22	50.36 ± 5.42	0.325	0.872
Handedness	18R	18 R	N/A	>0.99
Duration (years)	1.12 ± 0.32	N/A	N/A	N/A
Best-corrected VA-L	1.00 ± 0.10	1.05 ± 0.15	0.989	0.121
Best-corrected VA-R	1.05 ± 0.15	0.95 ± 0.10	0.086	0.127
IOP-L	27.56 ± 6.67	15.69 ± 4.86	12.087	0.012
IOP-R	27.61 ± 5.32	14.78 ± 4.39	11.537	0.009

*Independent t–tests comparing the age of two groups (P <0.05) represented statistically significant differences). Male/female and Handedness were analyzed using chi-squared test*.

*OH, ocular hypertension; HCs, healthy control; N/A, not applicable; VA, visual acuity; R, right; L, left; IOP, intraocular pressure*.

### The Manifestation of fALFF

**Figure 2:**
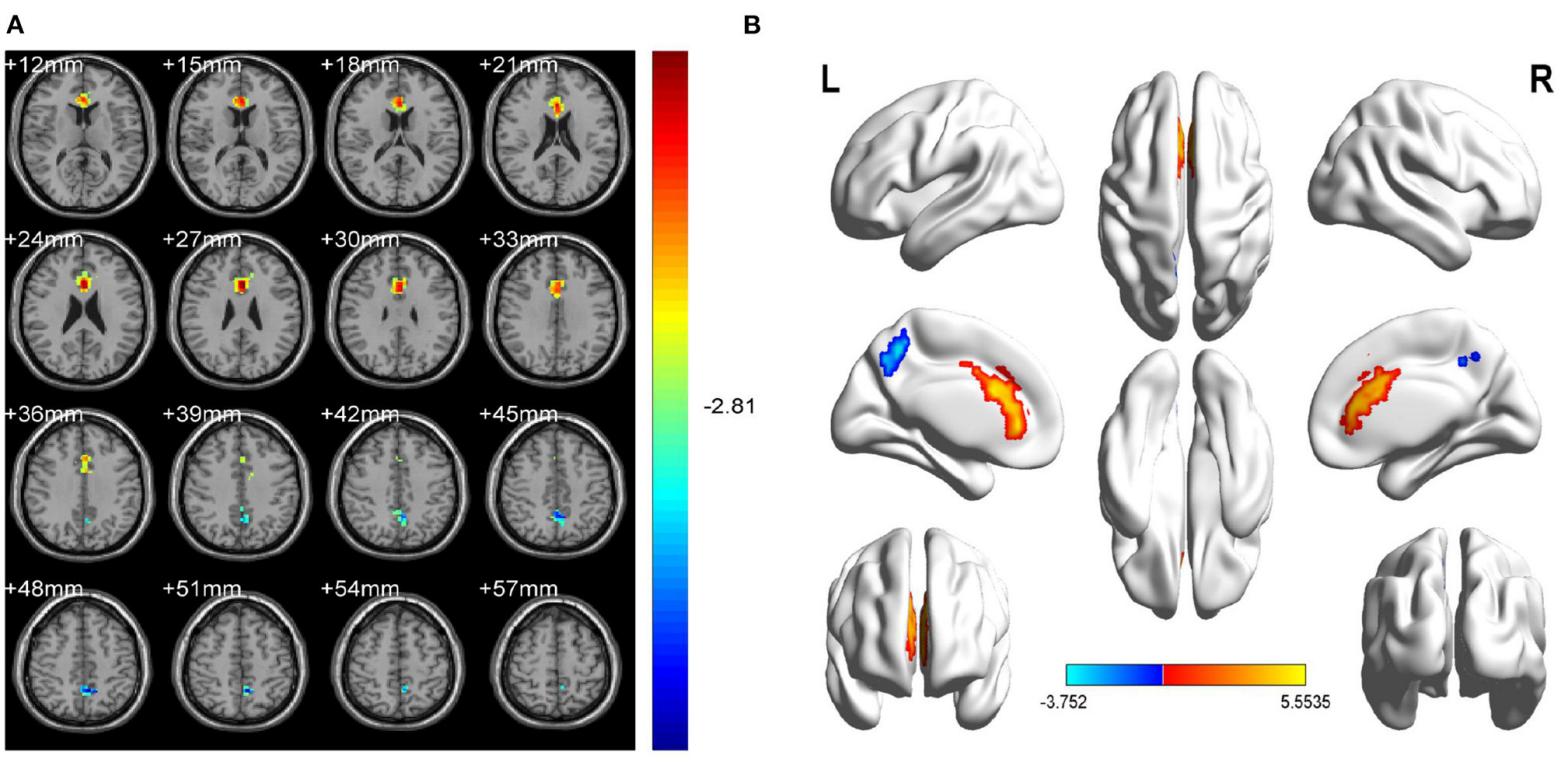
Spontaneous brain activity in OH vs. HC. **(A)** Different fALFF values between the OH and HC groups. **(B)** Differences of brain activity in the cerebrum. Compared with HC, red represents the brain areas with increased fALFF, and blue represents the brain areas with decreased fALFF in OH patients. Multiple comparisons were corrected using Gaussian Random Field (GFR) theory (two-tailed, voxel-level *P* < 0.01, cluster-level *P* < 0.05). fALFF, fractional amplitude of low-frequency fluctuations; ALFF, amplitude of low-frequency fluctuation; OH, ocular hypertension; HC, healthy control; R, right; L, left.

**Figure 3:**
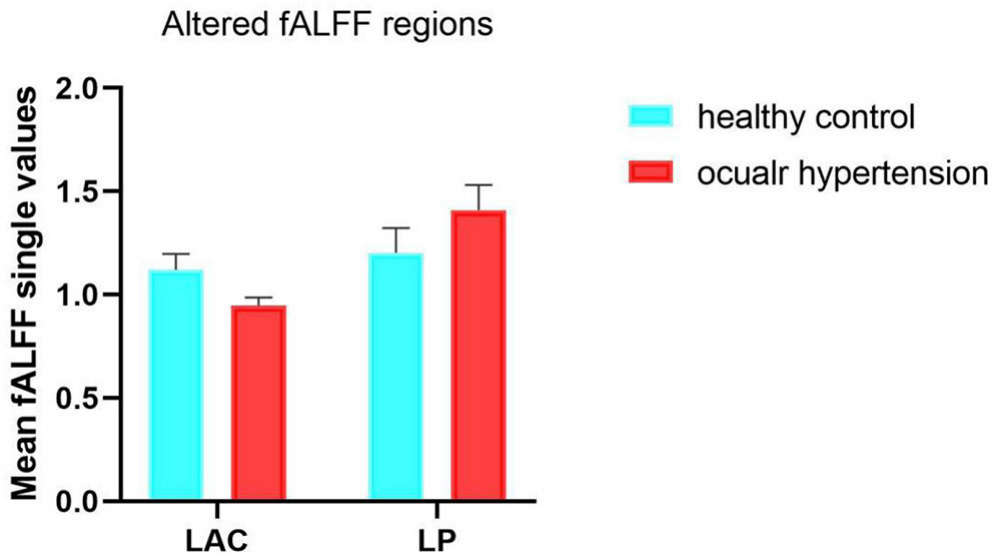
Mean ALFF values between the OH and HC groups in the different regions of the brain. fALFF, fractional amplitude of low-frequency fluctuations; ALFF, amplitude of low-frequency fluctuations; OH, ocular hypertension; HC, healthy control; LAC, left anterior cingulate; LP, left precuneus.

Mean fALFF value in the left precuneus (LP) was significantly higher in the OH group than in controls (*P* < 0.05; Table 3; Figure 2). Conversely, in the left anterior cingulate gyrus (LAC) the value was significantly lower in the OH group (Table 3; Figure 2). Figure 3 shows differences in mean fALFF value between the OH and HC groups. In each of the two groups, mean fALFF values in different brain regions were correlated with their clinical manifestations (*P* > 0.05).

**Table 3:** Compared with HC group, the fALFF values in OH group.

**Brain areas**	**MNI coordinates**			**BA**	**Number of voxels**	**T-value**
	**X**	**Y**	**Z**			
**HC** **>** **OH**
LAC	−3	18	27	31	341	5.5535
**HC** **<** **OH**
LP	−6	−51	51	67	95	−3.752

*Multiple comparisons were corrected using Gaussian Random Field (GFR)theory (two-tailed, voxel-level P <0.01, cluster-level P <0.05)*.

*HC, Healthy control; OH:Ocular hypertension;MNI, Montreal Neurological Institute; BA, Brodmann Area; LAC, left anterior cingulate; LP, left precuneus*.

### The Use of ROC

**Figure 4:**
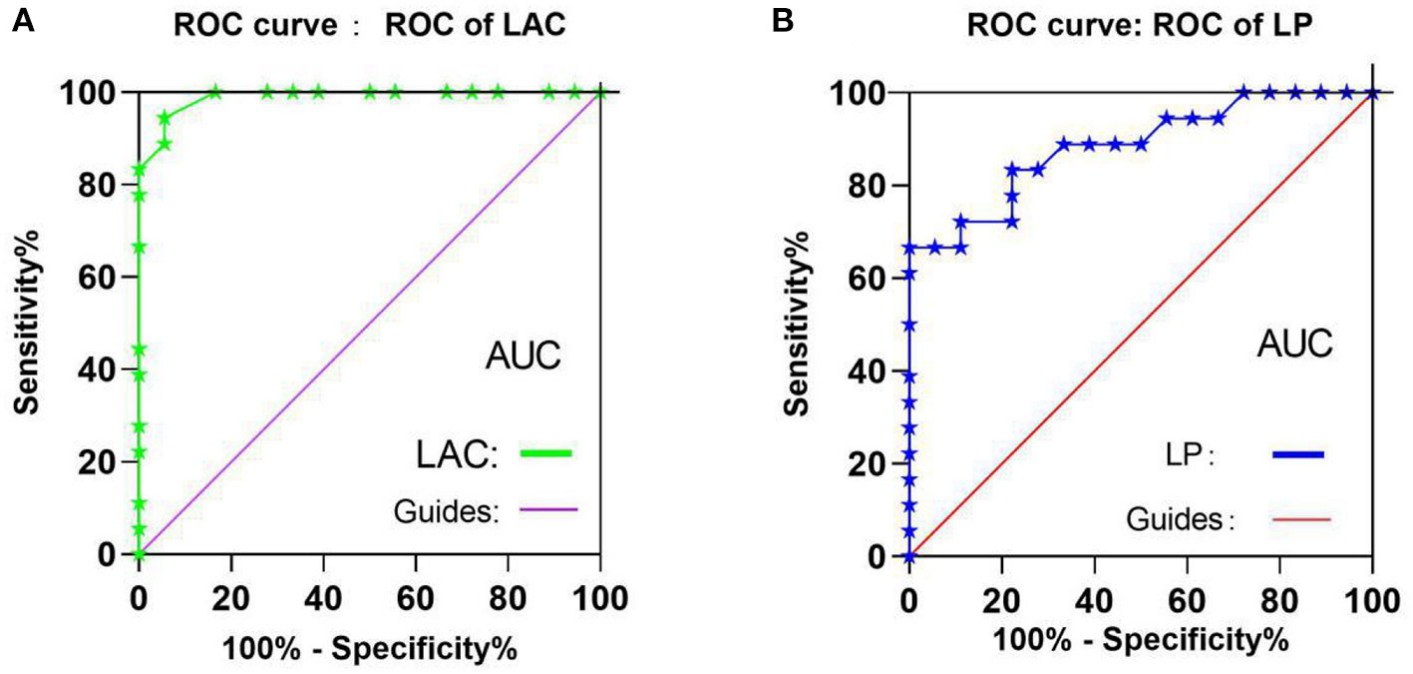
ROC curve analysis of the mean fALFF values of the two brain regions in two groups. **(A)** The areas under the ROC curve were: LAC 0.990, (*P* < 0.001; 95%CI,0.967-1.000), **(B)** The areas under the ROC curve were: LP 0.884 (*P* < 0.001; 95%CI,0.775-0.994). ROC, receiver operating characteristic; fALFF, fractional amplitude of low-frequency fluctuations; ALFF, amplitude of low-frequency fluctuation; CI, confidence interval; AUC, area under the curve;LAC, left anterior cingulate; LP, left precuneus.

To test whether the fALFF value has potential as a diagnostic marker for OH, ROC curves were constructed using mean fALFF values of the two brain regions in the OH and HC groups. An area under the curve of 0.5–0.7 is considered to indicate low accuracy, 0.7–0.9 higher accuracy, and an area higher than 0.9 represents good accuracy. Accuracy was high in the left anterior cingulate gyrus (LAC) (0.9892; *P* < 0.0001; Figure 4A) and lower in the left precuneus (LP) (0.8834; *P* < 0.0001; Figure 4B).

### The fALFF Value

**Figure 5:**
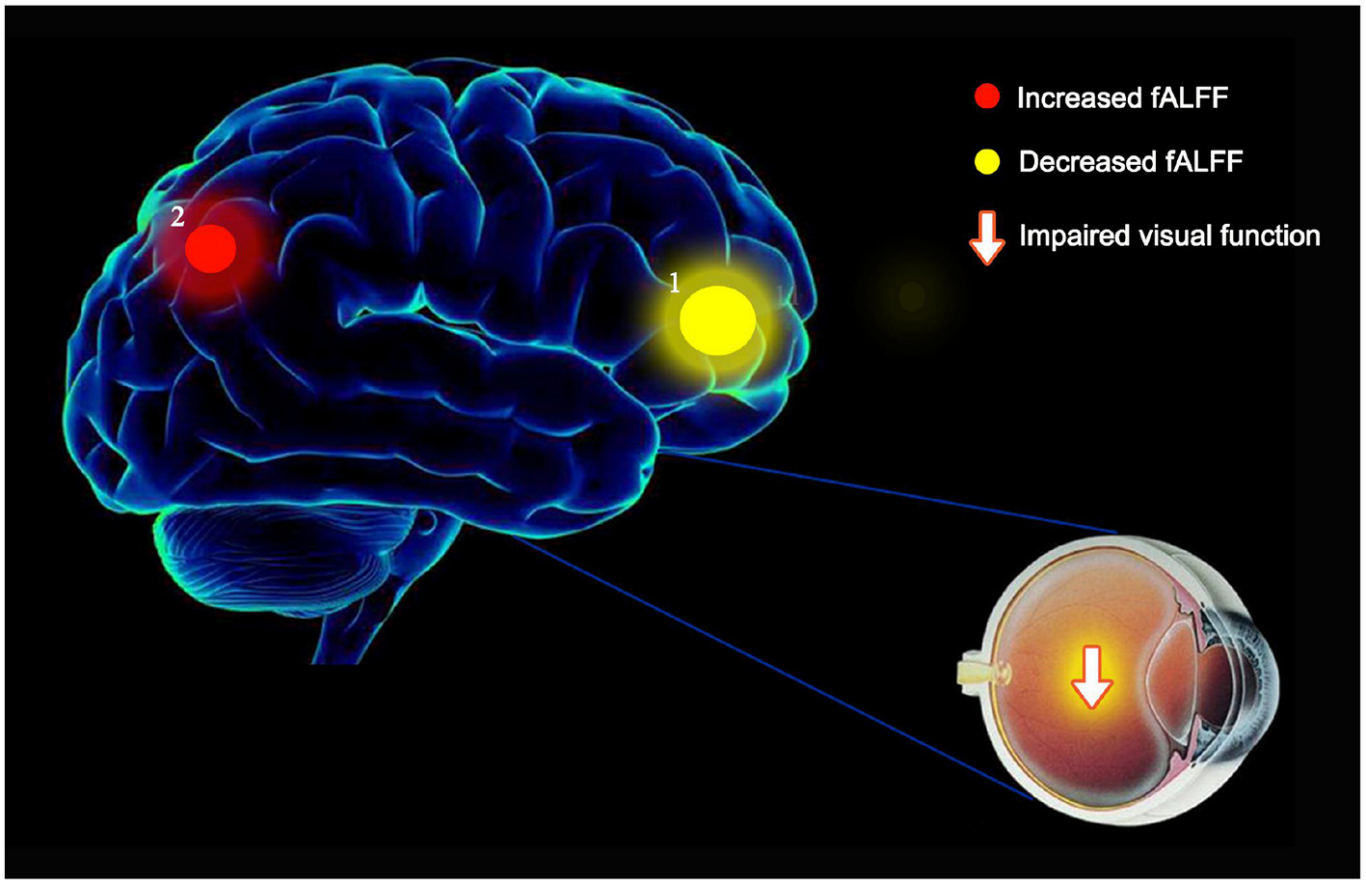
The mean fALFF values of altered brain regions in the OH group. (1) Compared with the HC, the fALFF values of the following regions were decreased to various extents: LAC (BA1, *t* = 5.5535). (2) Compared with the HC, the fALFF values of the following regions were increased to various extents: LP (BA2, *t* = −3.7520). fALFF, fractional amplitude of low-frequency fluctuations; ALFF, amplitude of low-frequency fluctuations; OH, ocular hypertension; HC, healthy control; R, right; L, left; BA, Brodmann's area; LAC, left anterior cingulate; LP, left precuneus.

The fALFF value of LP in the OH group was higher than in the HC group (*t* = –3.7520); while the fALFF value of LAC in the OH group was decreased (*t* = 5.5535) Figure 5).

## Discussion

The etiology and mechanism of OH are not well understood. This study is the first to use the fALFF method to research activity patterns in different brain regions in OH patients (Figure 5). Compared with healthy volunteers with normal IOP, the fALFF value of the left anterior cuneiform lobe of patients with OH was increased, while activity in the anterior cingulate gyrus on the ipsilateral side was decreased. These differences may be related to the functions of different brain regions.

Studies have shown that anterior cingulate gyrus and cuneiform are both indispensable components of the default-mode network (DMN), but they have both division of labor and cooperation in function (54, 55). As a part of the occipital lobe, the cuneiform lobe includes two important areas: 7M and precuneus vision (PCV) (56, 57). They are involved in visual spatial processing, contextual memory, and self-awareness in the visual system (58). Li et al. found that the default mode network (DMN) and visual cortex of patients with Primary Open-Angle Glaucoma (POAG) were significantly reduced in the fALFF value. Through the measurement table, they also found the lower the ALFF value, the more severe the disease is (41). In another study for NTG, an important subtype of POAG, which was found that the average fALFF value of the patient's right precuneus was reduced. At the same time, the fALFF value is opposite to the thickness of the retinal nerve fiber layer (RNFL) of the patients (40). In addition, in PACG patients, the left cuneus had significant lower fALFF values, but the data of this study showed that the change direction of average fALFF value of the left anterior cuneus was as the same as the trend of the patients' MDVF and RNFL thickness changes (42). In a half-year study on the adaptation of the optic nerve after cataract intraocular lens implantation, it was found that the increased fALFF value represented the brain's activation of the optic nerve adaptation mechanism: the decreased fALFF value suggested the adaptability of patient's visual function is inhibited, and the fALFF value of patients in the same group could changes from low to high or from high to low at different stages of visual function adaptation (59). Our current study found that in OH patients, the average fALFF value of the LP area was higher than that of the Hc group, which is the same as the conclusion drawn by the study of RP (36) and NVG (43). Therefore, we speculate that the intraocular pressure of patients with OH that exceeds the upper limit of the statistical value is an external manifestation of changes in brain function. The increase in the fALFF value of LP represents the activation of this brain area to adapt to visual changes. The role of maintaining normal visual function, and when OH progresses to glaucoma, such as POAG, the role of this activation adaptive mechanism is hindered, so that is why the fALFF value of the cuneiform lobe in glaucoma patients is significantly reduced, which perfectly meet the current view that ocular hypertension is regarded as a pre-glaucoma state (8, 9).

There may be two explanations for the abnormal changes of fALFF value in the two brain area of OH patients: first, it is a mechanism similar to compensatory recruitment. Due to the stimulation of the system by high intraocular pressure, the LAC area is in the adaptive compensation phase or a kind of reserve activation stage, at the same time, the LP area is in an active stage. The activities of these two areas try to achieve the purpose of adaptation through such adjustment and compensation, so that the neural network works normally; second, it reflects the plasticity of the brain, assuming that the LAC area arise neurodegenerative in the central nervous system of OH patients, but the LP area exhibits selective plasticity repair to maintain the balance. When OH has got into POAG, these two mechanisms have broken and undergone complex changes, then the activities of cuneiform and cingulate gyrus of OH correspond to the opposite in POAG. It is hard to propose whether OH and POAG disease progression is inevitable related to the changes in the fALFF value of the brain area because of differences in diseases, but if rs-MRI scans can be added to the indicator monitoring of the OH population, taking fALFF value as a reliable biological markers and continue to track them along the progression of OH to glaucoma. This will be a direction worthy of further exploration and research.

### Limitations

The potential limitations of this research include, first, the relatively small number of patients, which may have led to the lack of linear correlation of responses within each brain region between the two groups. Despite this weakness, other differences between the OH group and HC group were statistically significant. Second, the research is hospital based, and may be confounded by selection bias. Third, some of the differences observed between the study group and the control group were only apparent on one side of the brain. Future research may explore whether differences elsewhere will become significant with a larger number of research participants, or whether this is a biological phenomenon. Fourth, the study was limited by the currently available imaging technology, Many phenomena have not been explained strongly enough. For example, patients with POAG and PACG are prone to negative feelings such as eye pain or ipsilateral headache when the intraocular pressure is increased. Research has found that discomfort such as pain can induce the synaptic plasticity of ACC. Thereby enhancing the response to sensory stimuli (60, 61), which may be one of the factors that affect their response to the cingulate gyrus, but this evidence is not strong enough, because NVG patients may also have similar symptoms, however, the response to the cingulate gyrus of NVG was reduced. Regrettably, neither the previous studies of other diseases by scholars nor our current study have evaluated and compared the pain value of patients. Therefore, based on the existing research, the bias caused by pain on the research cannot be ruled out for the time being, which will be further updated and more powerful in combination with evaluation factors such as pain and depression in the future.

## Conclusion

In conclusion, our current study confirmed that there are abnormal neural activities in the LP and LAC region of the brain of OH patients, which not only allows ophthalmologists to diagnose OH faster and more accurately, but also offers a new direction for distinguishing patients with OH from PAOG. Most importantly, fALFF could be used as a sensitive biomarker for monitoring the course of OH.

## Author's Note

Our research aims to use the fractional amplitude of low-frequency fluctuation method to compare the performance characteristics of the same brain regions in patients with ocular hypertension and healthy volunteers under resting-state functional magnetic resonance. Our current research has confirmed that there are changes in fALFF values in two brain regions related to vision: the left precuneus lobe (LP) and the left anterior cingulate lobe (LAC). This not only shows that although patients with ocular hypertension have not experienced significant changes in the fundus for the time being, they still need to cooperate with regular review and close follow-up. It also provides reliable evidence and effective ideas for the pathogenesis of the disease at the level of the central nervous system.

## Data Availability Statement

The raw data supporting the conclusions of this article will be made available by the authors, without undue reservation.

## Ethics Statement

The studies involving human participants were reviewed and approved by the First Affiliated Hospital of Nanchang University. The patients/participants provided their written informed consent to participate in this study. Written informed consent was obtained from the individual(s) for the publication of any potentially identifiable images or data included in this article.

## Author Contributions

YL wrote this article. Y-CP processed the data. H-LL and YS reviewed all the work, and other authors participated in the whole work. All authors listed have made a substantial, direct, and intellectual contribution to the work and approved it for publication.

## Funding

This work was supported by Key Research Foundation of Jiangxi Province (No: 20181BBG70004); Excellent Talents Development Project of Jiangxi Province (20192BCBL23020); Natural Science Foundation of jiangxi Province (20181BAB205034); Grassroots Health Appropriate Technology Spark Promotion Plan Project of Jiangxi Province (No:20188003); Health Development Planning Commission Science Foundation of Jiangxi Province (No: 20201032); Health Development Planning Commission Science TCM Foundation of Jiangxi Province (No: 2018A060).

## Conflict of Interest

The authors declare that the research was conducted in the absence of any commercial or financial relationships that could be construed as a potential conflict of interest.

## Publisher's Note

All claims expressed in this article are solely those of the authors and do not necessarily represent those of their affiliated organizations, or those of the publisher, the editors and the reviewers. Any product that may be evaluated in this article, or claim that may be made by its manufacturer, is not guaranteed or endorsed by the publisher.

## References

[1] Levine RA Demirel S Fan J Keltner JL Johnson CA Kass MA. Asymmetries and visual field summaries as predictors of glaucoma in the ocular hypertension treatment study. Invest Ophthalmol Vis Sci. (2006) 47:3896–903. 10.1167/iovs.05-0469 16936102 PMC1995656

[2] Tham YC Li X Wong TY Quigley HA Aung T Cheng CY. Global prevalence of glaucoma and projections of glaucoma burden through 2040: a systematic review and meta-analysis. Ophthalmology. (2014) 121:2081–90. 10.1016/j.ophtha.2014.05.013 24974815

[3] Ge J Sun XH Wang NL Zhao JL Wu LL Chen XM. Intraocular pressure lowering efficacy and safety of travoprost 0.004% as a replacement therapy in patients with open angle glaucoma or ocular hypertension. Chin Med J. (2010) 123:1417–21. 10.3760/cma.j.issn.0366-6999.2010.11.012 20819599

[4] Xie X Chen W Li Z Thomas R Li Y Xian J. Noninvasive evaluation of cerebrospinal fluid pressure in ocular hypertension: a preliminary study. Acta Ophthalmol. (2018) 96:e570–6. 10.1111/aos.13724 29575652

[5] Varma R Ying-Lai M Francis BA Nguyen BB Deneen J Wilson MR. Prevalence of open-angle glaucoma and ocular hypertension in Latinos: the Los Angeles Latino Eye Study. Ophthalmology. (2004) 111:1439–48. 10.1016/j.ophtha.2004.01.025 15288969

[6] Rossi GC Pasinetti GM Scudeller L Raimondi M Lanteri S Bianchi PE. Risk factors to develop ocular surface disease in treated glaucoma or ocular hypertension patients. Eur J Ophthalmol. (2013) 23:296–302. 10.5301/ejo.5000220 23335308

[7] Ocular Hypertension Treatment Study Group European Glaucoma Prevention Study Group Gordon MO Torri V Miglior S Beiser JA. Validated prediction model for the development of primary open-angle glaucoma in individuals with ocular hypertension. Ophthalmology. (2007) 114:10–9. 10.1016/j.ophtha.2006.08.031 17095090 PMC1995665

[8] Gordon MO Kass MA. What we have learned from the ocular hypertension treatment study. Am J Ophthalmol. (2018) 189:xxiv–ii. 10.1016/j.ajo.2018.02.016 29501371 PMC5915899

[9] Giorgio A Zhang J Costantino F De Stefano N Frezzotti P. Altered large-scale brain functional connectivity in ocular hypertension. Front Neurosci. (2020) 14:146. 10.3389/fnins.2020.00146 32194370 PMC7066214

[10] Engelhorn T Michelson G Waerntges S Struffert T Haider S Doerfler A. Diffusion tensor imaging detects rarefaction of optic radiation in glaucoma patients. Acad Radiol. (2011) 18:764–9. 10.1016/j.acra.2011.01.014 21377906

[11] Engelhorn T Michelson G Waerntges S Hempel S El-Rafei A Struffert T. A new approach to assess intracranial white matter abnormalities in glaucoma patients: changes of fractional anisotropy detected by 3T diffusion tensor imaging. Acad Radiol. (2012) 19:485–8. 10.1016/j.acra.2011.12.005 22277635

[12] Shimazawa M Ito Y Inokuchi Y Yamanaka H Nakanishi T Hayashi T. An alteration in the lateral geniculate nucleus of experimental glaucoma monkeys: *in vivo* positron emission tomography imaging of glial activation. PLoS ONE. (2012) 7:e30526. 10.1371/journal.pone.0030526 22299044 PMC3267730

[13] Michelson G Engelhorn T Wärntges S El Rafei A Hornegger J Doerfler A. DTI parameters of axonal integrity and demyelination of the optic radiation correlate with glaucoma indices. Graefes Arch Clin Exp Ophthalmol. (2013) 251:243–53. 10.1007/s00417-011-1887-2 22366916

[14] Chen WW Wang N Cai S Fang Z Yu M Wu Q. Structural brain abnormalities in patients with primary open-angle glaucoma: a study with 3T MR imaging. Invest Ophthalmol Vis Sci. (2013) 54:545–54. 10.1167/iovs.12-9893 23258150

[15] Zhang YQ Li J Xu L Zhang L Wang ZC Yang H. Anterior visual pathway assessment by magnetic resonance imaging in normal-pressure glaucoma. Acta Ophthalmol. (2012) 90:e295–302. 10.1111/j.1755-3768.2011.02346.x 22489916

[16] Li C Cai P Shi L Lin Y Zhang J Liu S. Voxel-based morphometry of the visual-related cortex in primary open angle glaucoma. Curr Eye Res. (2012) 37:794–802. 10.3109/02713683.2012.683506 22631870

[17] Williams AL Lackey J Wizov SS Chia TM Gatla S Moster ML. Evidence for widespread structural brain changes in glaucoma: a preliminary voxel-based MRI study. Invest Ophthalmol Vis Sci. (2013) 54:5880–7. 10.1167/iovs.13-11776 23838767

[18] Wang J Li T Sabel BA Chen Z Wen H Li J. Structural brain alterations in primary open angle glaucoma: a 3T MRI study. Sci Rep. (2016) 6:18969. 10.1038/srep18969 26743811 PMC4705520

[19] Tehrani S. Gender difference in the pathophysiology and treatment of glaucoma. Curr Eye Res. (2015) 40:191–200. 10.3109/02713683.2014.968935 25285808

[20] Kim JH Caprioli J. Intraocular pressure fluctuation: is it important? J Ophthalmic Vis Res. (2018) 13:170–4. 10.4103/jovr.jovr_35_18 29719646 PMC5905311

[21] Nuzzi R Dallorto L Rolle T. Changes of visual pathway and brain connectivity in glaucoma: a systematic review. Front Neurosci. (2018) 12:363. 10.3389/fnins.2018.00363 29896087 PMC5986964

[22] Liu XY Chen XM Wang NL. [Is glaucoma a central nervous system disease: re-evaluation]. Zhonghua Yan Ke Za Zhi. (2010) 46:1062–5. 10.3760/cma.j.issn.0412-4081.2010.12.003 21211216

[23] Dai H Mu KT Qi JP Wang CY Zhu WZ Xia LM. Assessment of lateral geniculate nucleus atrophy with 3T MR imaging and correlation with clinical stage of glaucoma. AJNR Am J Neuroradiol. (2011) 32:1347–53. 10.3174/ajnr.A2486 21757515 PMC7966066

[24] Maknojia S Churchill NW Schweizer TA Graham SJ. Resting state fMRI: going through the motions. Front Neurosci. (2019) 13:825. 10.3389/fnins.2019.00825 31456656 PMC6700228

[25] Collins DL Zijdenbos AP Kollokian V Sled JG Kabani NJ Holmes CJ. Design and construction of a realistic digital brain phantom. IEEE Trans Med Imaging. (1998) 17:463–8. 10.1109/42.712135 9735909

[26] Kiviniemi V Kantola JH Jauhiainen J Tervonen O. Comparison of methods for detecting nondeterministic BOLD fluctuation in fMRI. Magn Reson Imaging. (2004) 22:197–203. 10.1016/j.mri.2003.09.007 15010111

[27] Wang Y Lu W Yan T Zhou J Xie Y Yuan J. Functional MRI reveals effects of high intraocular pressure on central nervous system in high-tension glaucoma patients. Acta Ophthalmol. (2019) 97:e341–8. 10.1111/aos.14027 30801975

[28] Zang YF He Y Zhu CZ Cao QJ Sui MQ Liang M. Altered baseline brain activity in children with ADHD revealed by resting-state functional MRI. Brain Dev. (2007) 29:83–91. 10.1016/j.braindev.2006.07.002 16919409

[29] Huang X Zhou FQ Hu YX Xu XX Zhou X Zhong YL. Altered spontaneous brain activity pattern in patients with high myopia using amplitude of low-frequency fluctuation: a resting-state fMRI study. Neuropsychiatr Dis Treat. (2016) 12:2949–56. 10.2147/NDT.S118326 27881920 PMC5115684

[30] Liu Z Tian J. Amplitude of low frequency fluctuation in primary open angle glaucoma: a resting state fMRI study. Annu Int Conf IEEE Eng Med Biol Soc. (2014) 2014:6706–9. 10.1109/EMBC.2014.6945167 25571535

[31] Li Q Huang X Ye L Wei R Zhang Y Zhong YL. Altered spontaneous brain activity pattern in patients with late monocular blindness in middle-age using amplitude of low-frequency fluctuation: a resting-state functional MRI study. Clin Interv Aging. (2016) 11:1773–80. 10.2147/CIA.S117292 27980398 PMC5147398

[32] Huang X Cai FQ Hu PH Zhong YL Zhang Y Wei R. Disturbed spontaneous brain-activity pattern in patients with optic neuritis using amplitude of low-frequency fluctuation: a functional magnetic resonance imaging study. Neuropsychiatr Dis Treat. (2015) 11:3075–83. 10.2147/NDT.S92497 26719692 PMC4689287

[33] Shi WQ Tang LY Lin Q Li B Jiang N Zhu PW. Altered spontaneous brain activity patterns in diabetic patients with vitreous hemorrhage using amplitude of low-frequency fluctuation: a resting-state fMRI study. Mol Med Rep. (2020) 22:2291–9. 10.3892/mmr.2020.11294 32705185 PMC7411342

[34] Wu YY Yuan Q Li B Lin Q Zhu PW Min YL. Altered spontaneous brain activity patterns in patients with retinal vein occlusion indicated by the amplitude of low-frequency fluctuation: a functional magnetic resonance imaging study. Exp Ther Med. (2019) 18:2063–71. 10.3892/etm.2019.7770 31410162 PMC6676080

[35] Chen W Wu Q Chen L Zhou J Chen HH Xu XQ. Disrupted spontaneous neural activity in patients with thyroid-associated ophthalmopathy: a resting-state fMRI study using amplitude of low-frequency fluctuation. Front Hum Neurosci. (2021) 15:676967. 10.3389/fnhum.2021.676967 34177495 PMC8226248

[36] Huang X Zhou FQ Dan HD Shen Y. Abnormal intrinsic brain activity in individuals with peripheral vision loss because of retinitis pigmentosa using amplitude of low-frequency fluctuations. Neuroreport. (2018) 29:1323–32. 10.1097/WNR.0000000000001116 30113921

[37] Chen J Jin H Zhong YL Huang X. Abnormal low-frequency oscillations reflect abnormal eye movement and stereovision in patients with comitant exotropia. Front Hum Neurosci. (2021) 15:754234. 10.3389/fnhum.2021.754234 34690728 PMC8531266

[38] Zou QH Zhu CZ Yang Y Zuo XN Long XY Cao QJ. An improved approach to detection of amplitude of low-frequency fluctuation (ALFF) for resting-state fMRI: fractional ALFF. J Neurosci Methods. (2008) 172:137–41. 10.1016/j.jneumeth.2008.04.012 18501969 PMC3902859

[39] Duncan RO Sample PA Weinreb RN Bowd C Zangwill LM. Retinotopic organization of primary visual cortex in glaucoma: comparing fMRI measurements of cortical function with visual field loss. Prog Retin Eye Res. (2007) 26:38–56. 10.1016/j.preteyeres.2006.10.001 17126063 PMC1940234

[40] Li HL Chou XM Liang Y Pan T Zhou Q Pei CG. Use of rsfMRI-fALFF for the detection of changes in brain activity in patients with normal-tension glaucoma. Acta Radiol. (2021) 62:414–22. 10.1177/0284185120926901 32571098

[41] Li T Liu Z Li J Liu Z Tang Z Xie X. Altered amplitude of low-frequency fluctuation in primary open-angle glaucoma: a resting-state fMRI study. Invest Ophthalmol Vis Sci. (2014) 56:322–9. 10.1167/iovs.14-14974 25525176

[42] Wang R Tang Z Liu T Sun X Wu L Xiao Z. Altered spontaneous neuronal activity and functional connectivity pattern in primary angle-closure glaucoma: a resting-state fMRI study. Neurol Sci. (2021) 42:243–51. 10.1007/s10072-020-04577-1 32632634

[43] Zhang YQ Peng MY Wu SN Yu CY Chen SY Tan SW. Fractional amplitude of low-frequency fluctuation in patients with neovascular glaucoma: a resting-state functional magnetic resonance imaging study. Quant Imaging Med Surg. (2021) 11:2138–50. 10.21037/qims-20-855 33936994 PMC8047371

[44] Fang JW Yu YJ Tang LY Chen SY Zhang MY Sun T. Abnormal fractional amplitude of low-frequency fluctuation changes in patients with monocular blindness: a functional magnetic resonance imaging (MRI) study. Med Sci Monit. (2020) 26:e926224. 10.12659/MSM.926224 32773731 PMC7439597

[45] Feng YX Li RY Wei W Feng ZJ Sun YK Sun HY. The acts of opening and closing the eyes are of importance for congenital blindness: evidence from resting-state fMRI. Neuroimage. (2021) 233:117966. 10.1016/j.neuroimage.2021.117966 33744460

[46] Chen Q Zheng W Chen X Li X Wang L Qin W. Whether visual-related structural and functional changes occur in brain of patients with acute incomplete cervical cord injury: a multimodal based MRI study. Neuroscience. (2018) 393:284–94. 10.1016/j.neuroscience.2018.10.014 30326291

[47] Yang L Yan Y Li Y Hu X Lu J Chan P. Frequency-dependent changes in fractional amplitude of low-frequency oscillations in Alzheimer's disease: a resting-state fMRI study. Brain Imaging Behav. (2020) 14:2187–201. 10.1007/s11682-019-00169-6 31478145

[48] Puche Sarmiento AC Bocanegra García Y Ochoa Gómez JF. Active information storage in Parkinson's disease: a resting state fMRI study over the sensorimotor cortex. Brain Imaging Behav. (2020) 14:1143–53. 10.1007/s11682-019-00037-3 30684153

[49] Seidel M Borchardt V Geisler D King JA Boehm I Pauligk S. Abnormal spontaneous regional brain activity in young patients with anorexia nervosa. J Am Acad Child Adolesc Psychiatry. (2019) 58:1104–14. 10.1016/j.jaac.2019.01.011 30768380

[50] Sun N Li Y Zhang A Yang C Liu P Liu Z. Fractional amplitude of low-frequency fluctuations and gray matter volume alterations in patients with bipolar depression. Neurosci Lett. (2020) 730:135030. 10.1016/j.neulet.2020.135030 32389612

[51] Ao W Cheng Y Chen M Wei F Yang G An Y. Intrinsic brain abnormalities of irritable bowel syndrome with diarrhea: a preliminary resting-state functional magnetic resonance imaging study. BMC Med Imaging. (2021) 21:4. 10.1186/s12880-020-00541-9 33407222 PMC7788841

[52] Piao S Wang R Qin H Hu B Du J Wu H. Alterations of spontaneous brain activity in systematic lupus erythematosus patients without neuropsychiatric symptoms: a resting-functional MRI study. Lupus. (2021) 30:1781–9. 10.1177/09612033211033984 34620007

[53] Chang J Yu R. Acute social stress modulates coherence regional homogeneity. Brain Imaging Behav. (2019) 13:762–70. 10.1007/s11682-018-9898-9 29802600

[54] Tolomeo S Christmas D Jentzsch I Johnston B Sprengelmeyer R Matthews K. A causal role for the anterior mid-cingulate cortex in negative affect and cognitive control. Brain. (2016) 139:1844–54. 10.1093/brain/aww069 27190027

[55] Zhang WN Chang SH Guo LY Zhang KL Wang J. The neural correlates of reward-related processing in major depressive disorder: a meta-analysis of functional magnetic resonance imaging studies. J Affect Disord. (2013) 151:531–9. 10.1016/j.jad.2013.06.039 23856280

[56] Baker CM Burks JD Briggs RG Conner AK Glenn CA Manohar K. A connectomic atlas of the human cerebrum-chapter 8: the posterior cingulate cortex, medial parietal lobe, and parieto-occipital sulcus. Oper Neurosurg. (2018) 15:S350–71. 10.1093/ons/opy262 30260425 PMC6887737

[57] Cavanna AE Trimble MR. The precuneus: a review of its functional anatomy and behavioural correlates. Brain. (2006) 129:564–83. 10.1093/brain/awl004 16399806

[58] Paulsen DJ Hallquist MN Geier CF Luna B. Effects of incentives, age, and behavior on brain activation during inhibitory control: a longitudinal fMRI study. Dev Cogn Neurosci. (2015) 11:105–15. 10.1016/j.dcn.2014.09.003 25284272 PMC4323861

[59] Zhang L Lin D Wang Y Chen W Xiao W Xiang Y. Comparison of visual neuroadaptations after multifocal and monofocal intraocular lens implantation. Front Neurosci. (2021) 15:648863. 10.3389/fnins.2021.648863 34194292 PMC8236945

[60] Wei F Zhuo M. Potentiation of sensory responses in the anterior cingulate cortex following digit amputation in the anaesthetised rat. J Physiol. (2001) 532:823–33. 10.1111/j.1469-7793.2001.0823e.x 11313449 PMC2278568

[61] Bliss TV Collingridge GL Kaang BK Zhuo M. Synaptic plasticity in the anterior cingulate cortex in acute and chronic pain. Nat Rev Neurosci. (2016) 17:485–96. 10.1038/nrn.2016.68 27307118

