# Corrigendum: Characteristics of the Fractional Amplitude of Low-Frequency Fluctuation in Ocular Hypertension Patients: A Resting-State fMRI Study

**DOI:** 10.3389/fmed.2022.958937

**Published:** 2022-06-29

**Authors:** Ying Liang, Yi-Cong Pan, Hui-Ye Shu, Xue-Mei Chou, Qian-Min Ge, Li-Juan Zhang, Qiu-Yu Li, Rong-Bing Liang, Han-Lin Li, Yi Shao

**Affiliations:** Department of Ophthalmology, The First Affiliated Hospital of Nanchang University, Jiangxi Province Ophthalmology Institute and Ocular Disease Clinical Research Centre, Nanchang, China

**Keywords:** ocular hypertension, resting state functional magnetic resonance imaging, fractional amplitude of low-frequency fluctuation, left anterior cingulate cortex, left precuneus

In the original article, there was a mistake in Table 2. **Demographics and behavioral results of OH and HCs groups** as published. The age of HC was incorrectly listed as **50.36** **±** **54.22**. The correct age of HC is **50.36** **±** **5.42**. The corrected Table 2 appears below.

**Table 2:** Demographics and behavioral results of OH and HCs groups.

	**OH**	**HC**	***T*-value**	***P*-value**
Male/female	9/9	9/9	N/A	>0.99
Age (years)	51.18 ± 5.22	50.36 ± 5.42	0.325	0.872
Handedness	18R	18 R	N/A	>0.99
Duration (years)	1.12 ± 0.32	N/A	N/A	N/A
Best-corrected VA-L	1.00 ± 0.10	1.05 ± 0.15	0.989	0.121
Best-corrected VA-R	1.05 ± 0.15	0.95 ± 0.10	0.086	0.127
IOP-L	27.56 ± 6.67	15.69 ± 4.86	12.087	0.012
IOP-R	27.61 ± 5.32	14.78 ± 4.39	11.537	0.009

In the original article, there was an error in the section **Materials and Methods**, sub**-**section **Subjects**. The subject characteristics: “18 HC **patients** (**8** males and **8** females; mean age 50.36 ± **54.22** years; age range 20–60 years)” is incorrect. The correct sentence is: “18 HCs (9 males and 9 females; mean age 50.36 ± 5.42 years; age range 20–60 years)”.

In the original article, there was an error in the section **Results**, sub-section **The Use of ROC**. The sentence: “To test whether the **ROC curve** has potential as a diagnostic marker for OH, ROC curves were constructed using mean fALFF values of the two brain regions in the OH and HC groups. An area under the curve of 0.5–0.7 is considered to indicate low accuracy, 0.7–0.9 higher accuracy, and an area higher than 0.9 represents good accuracy. Accuracy was **low** in the left anterior cingulate gyrus (LAC) (0.9892; *P* < 0.0001; **Figure 4A**) and **higher** in the left **anterior cuneiform** (LP) **>** **HC** (0.8834; *P* < 0.0001; **Figure 4B**).” is incorrect. The sentence should be: “To test whether the fALFF value has potential as a diagnostic marker for OH, ROC curves were constructed using mean fALFF values of the two brain regions in the OH and HC groups. An area under the curve of 0.5–0.7 is considered to indicate low accuracy, 0.7–0.9 higher accuracy, and an area higher than 0.9 represents good accuracy. Accuracy was high in the left anterior cingulate gyrus (LAC) (0.9892; *P* < 0.0001; **Figure 4A**) and lower in the left precuneus (LP) (0.8834; *P* < 0.0001; **Figure 4B**).”

In the original article, there was an error in section **Results**, sub-section **The fALFF Value**. The values in the sentence: “The fALFF value of LP in the OH group was higher than in the HC group (***t* =** **5.5535**), while the fALFF value of LAC in the OH group was decreased (***t*** **=** **−3.7520**; **Figure 5**)” are incorrect. They should be: “The fALFF value of LP in the OH group was higher than in the HC group (*t* = −3.7520); while the fALFF value of LAC in the OH group was decreased (*t* = 5.5535) (**Figure 5**).”

The authors apologize for these errors and state that they do not change the scientific conclusions of the article in any way. The original article has been updated.

## Publisher's Note

All claims expressed in this article are solely those of the authors and do not necessarily represent those of their affiliated organizations, or those of the publisher, the editors and the reviewers. Any product that may be evaluated in this article, or claim that may be made by its manufacturer, is not guaranteed or endorsed by the publisher.

